# MicroRNA-377 Regulates Mesenchymal Stem Cell-Induced Angiogenesis in Ischemic Hearts by Targeting VEGF

**DOI:** 10.1371/journal.pone.0104666

**Published:** 2014-09-24

**Authors:** Zhili Wen, Wei Huang, Yuliang Feng, Wenfeng Cai, Yuhua Wang, Xiaohong Wang, Jialiang Liang, Mashhood Wani, Jing Chen, Pin Zhu, Ji-Mei Chen, Ronald W. Millard, Guo-Chang Fan, Yigang Wang

**Affiliations:** 1 Department of Infectious Disease, Nanchang University Medical School, Nanchang, Jiangxi, China; 2 Department of Pathology and Lab Medicine, University of Cincinnati Medical Center, Cincinnati, Ohio, United States of America; 3 Department of Pharmacology and Cell Biophysics, University of Cincinnati Medical Center, Cincinnati, Ohio, United States of America; 4 Department of Environmental Health, University of Cincinnati Medical Center, Cincinnati, Ohio, United States of America; 5 Guangdong Cardiovascular Institute, Guangdong Academy of Medical Sciences, Guangzhou, Guandong, People’s Republic of China; Georgia Regents University, United States of America

## Abstract

MicroRNAs have been appreciated in various cellular functions, including the regulation of angiogenesis. Mesenchymal-stem-cells (MSCs) transplanted to the MI heart improve cardiac function through paracrine-mediated angiogenesis. However, whether microRNAs regulate MSC induced angiogenesis remains to be clarified. Using microRNA microarray analysis, we identified a microRNA expression profile in hypoxia-treated MSCs and observed that among all dysregulated microRNAs, microRNA-377 was decreased the most significantly. We also validated that vascular endothelial growth factor (VEGF) is a target of microRNA-377 using dual-luciferase reporter assay and Western-blotting. Knockdown of endogenous microRNA-377 promoted tube formation in human umbilical vein endothelial cells. We then engineered rat MSCs with lentiviral vectors to either overexpress microRNA-377 (MSC^miR-377^) or knockdown microRNA-377 (MSC^Anti-377^) to investigate whether microRNA-377 regulated MSC-induced myocardial angiogenesis, using MSCs infected with lentiviral empty vector to serve as controls (MSC^Null^). Four weeks after implantation of the microRNA-engineered MSCs into the infarcted rat hearts, the vessel density was significantly increased in MSC^Anti-377^-hearts, and this was accompanied by reduced fibrosis and improved myocardial function as compared to controls. Adverse effects were observed in MSC^miR-377^-treated hearts, including reduced vessel density, impaired myocardial function, and increased fibrosis in comparison with MSC^Null^-group. These findings indicate that hypoxia-responsive microRNA-377 directly targets VEGF in MSCs, and knockdown of endogenous microRNA-377 promotes MSC-induced angiogenesis in the infarcted myocardium. Thus, microRNA-377 may serve as a novel therapeutic target for stem cell-based treatment of ischemic heart disease.

## Introduction

The formation of new blood vessels is critical for the repair of ischemic myocardium, and VEGF is one of the most extensively characterized angiogenic factors [1]. While direct administration of VEGF into the ischemic myocardium has been used successfully to stimulate therapeutic angiogenesis in animal models, clinical trials of VEGF have been largely unsuccessful [2], [3]. These results underscore our incomplete knowledge of myocardial angiogenesis under ischemic conditions.

During the past decade, it has been demonstrated that MSCs can facilitate new blood vessel growth by secretion of pro-angiogenic factors (e.g. VEGF, IGF-1α, HGF, etc.) that contribute to cardiac repair and enhance the reparative process [4]–[6]. MSCs are, however, highly sensitive to ischemic conditions, and the majority of injected MSCs die within several hours of delivery *in vivo* [7]. In this regard, multiple approaches (e.g. hypoxic treatment, genetic modification, and pre-conditioning) have been applied to MSCs in an effort to improve their survival and pro-angiogenic capacity both *in vivo* and *in vitro* [8]. Although hypoxia is well recognized to promote MSC-mediated myocardial angiogenesis by induction of VEGF expression [9], [10], the underlying mechanisms underlying these effects have not been delineated.

miRs are a class of 20–24 nt non-coding RNAs that negatively regulate protein-coding gene expression primarily through post-transcriptional repression or mRNA degradation in a sequence-specific manner [11]–[13]. Recently, miRs regulated by hypoxia have been profiled in endothelial cells (ECs) and cancer cells. For example, Kulshreshtha, *et al.* [14] identified that a set of miRs were consistently up-regulated in breast tumor and colon cancer cells in response to hypoxia. In addition, Fasanaro, *et al.* [15] first reported that hypoxia-driven miR-210 promotes angiogenic response in ECs by down-regulation of EFNA3, an ephrin family member involving vascular development. Recent studies also indicate that a panel of miRNAs (i.e., miR-10, miR-15b, miR-16, miR-20a, miR-20b, miR-27a, miR-126, miR-145, miR-195, miR-205, and miR-210) is involved in the regulation of VEGF expression in ECs and tumor cells [16]–[26]. Few studies however, have examined how hypoxia affects the miR expression profile in MSCs. In addition to further investigation of this process, a significant additional question for examination is whether hypoxia-associated miRs regulate MSC-induced myocardial angiogenesis in ischemic hearts.

In this study, we first sought to determine the alterations of miRs in MSCs under hypoxic conditions in rats. We then identified which hypoxia-inducible miRs could directly regulate VEGF expression and tested whether manipulation of such miR in MSCs could affect MSC-induced angiogenesis in ischemic myocardium. Our results show for the first time that miR-377 was strongly down-regulated in hypoxia-treated MSCs, which was a major factor contributing to the increased VEGF levels. Thus, injection of miR-377-knockdown MSCs into an infarcted myocardium reduced overall infarction size and improved contractile function by promoting angiogenesis. These findings suggest that miR-377 may serve as a novel potential therapeutic target for treatment of ischemic heart diseases.

## Materials and Methods

### Animal Experiments

All research protocols conformed to the Guidelines for the Care and Use of Laboratory Animals published by the National Institutes of Health (National Academies Press, 8^th^ edition, 2011). All animal use protocols and methods of euthanasia (pentobarbital overdose followed by thoracotomy) used in this study were approved by the University of Cincinnati Animal Care and Use Committee. The Institutional Biosafety Committee (IBC) conducted an independent review and approval of our cell and virus methods.

### *In Vitro* Studies

In order to culture MSCs, they were extracted from Sprague-Dawley (SD; 8-wk-old male) rats following previously published procedures from our laboratory [9]. MSCs were then cultured in Dulbecco’s Modified Eagle Medium(DMEM) supplemented with 10% (v/v) fetal bovine serum (FBS) and antibiotics (100 U/mL penicillin and 100 µg/mL streptomycin). The cells were kept in a humidified 5% CO_2_ incubator at 37°C and culture medium was changed after 3 days. Non-adherent cells were removed by changing the medium and the remaining adherent cells were primary MSCs. Passage 2–4 MSCs were used in this study.

Hypoxic MSCs were cultured in DMEM without glucose and with 1% FBS under hypoxic conditions of 1% O_2_, 5% CO_2_ and 94% N_2_ at 37°C in a hypoxic incubator (O_2_/CO_2_ incubator-MCO-18M; Sanyo) for 24 h. MSCs cultured in normal conditions (normoxia) served as a control.

### RNA Extraction and RT-PCR

Total RNA from the MSCs was extracted using the Trizol reagent (Invitrogen, Carlsbad, Calif., United States), as recommended by the manufacturer. Total RNA concentrations were determined by NanoVue plus (GE Healthcore, Piscataway, New Jersey, USA). The mRNA levels of VEGF and miRs were examined by reverse transcription-polymerase chain reaction (RT-PCR) or quantitative real-time PCR (qPCR), and β-Actin or U6 was used as an internal reference. The primers for VEGF and β-Actin were designed as follows: **VEGF** forward: 5′-GCAACACCAAGTCCGAATGCAGAT-3′, reverse: 5′-TCTGGCTTCACAGCACTCTCCTTT-3′; **β-Actin** forward: 5′-TGTGATGGTGGGAAT GGGTCAGAA-3′, reverse: 5′-TGTGGTGCCAGATCTTCTCCATGT-3′. The primers for miRNA and U6 were purchased from QIAGEN. The primers for miRs consisted of a specific primer (Rn_miR-377_2 miScript Primer Assay) and a universal primer (10× miScript Universal Primer). The amplification profiles for PCR: 94°C 5 min., followed by 30 cycles of 94°C 30 sec., 55°C 30 sec., 72°C 45 sec., and a final 5 min. extend; and for qPCR: 95°C 15 min., followed by 40 cycles of 94°C 15 sec., 55°C 30 sec., 72°C 30 sec. with 0.5°C/15 sec. in 55°C∼95°C. PCR products were analyzed with 1.5% agarose gel. The qPCR expression of VEGF mRNA relative to β-Actin under experimental and control conditions was calculated based on the threshold cycle (Ct) as n = 2^−Δ (ΔCt)^, where ΔCt = Ct _VEGF_ − Ct _β-Actin_ and Δ (ΔCt) = ΔCt experimental − ΔCt control. Individual experiments were repeated at least 3 times, and the *n*-mean value was calculated.

### Western-Blotting Analysis

Protein samples were collected from MSCs treated under different conditions, and 60 µg of protein was loaded and subjected to SDS-PAGE, as described previously [6]. PageRuler™ Plus Prestained Protein Ladder (Thermo Scientific Inc., MA, USA) was loaded as a protein marker to estimate molecular weight of samples. A VEGF antibody (Rabbit, 1∶500) was purchased from Santa Cruz Biotechnology. β-Actin (mouse 1∶1000) was purchased from Santa Cruz Biotechnology.

### miRNA Array Analysis and Target Prediction

Total RNA samples obtained from MSCs under normoxia or hypoxia were sent to LC Sciences (Houston, TX) for miRNA microarray profiling. Data was analyzed by LC Sciences with in-house developed computer programs. Intensity values were transformed into log2 scale, and fold changes were given in log2 scale. A *t*-test was performed between normoxic MSCs and hypoxic MSCs, and statistical significance was considered at *P*<0.01. The microarray data were confirmed using an miRNA detection protocol with RT^2^ miRNA First Strand Kit (SA biosciences). Computational miRNA target prediction analysis was performed with TargetScan (version 6.2) and miRDB to predict potential binding between VEGF 3′UTR and miRNA.

### Transient Transfection of MSCs with miRNA Mimic, miRNA Inhibitor or siRNA

miR-377 mimic and its negative control (NC) (miRIDIAN Mimic, Thermo Scientific), miR-377 inhibitor and its negative control (miRCURY LNA microRNA inhibitor, Exiqon), VEGF siRNA and its negative control(ON-TARGET plus SMARTpool, rat VEGF-A, Thermo Scientific) were assigned into 6 groups for transfection as follows: **A**. miR-377 mimic (miR-377); **B**. Negative control (NC)-miR-377-mimic (NC^miR^); **C**. miR-377 inhibitor (Anti-377); **D**. NC-miR-377-inhibitor (NC^Anti-377^); **E**. VEGF siRNA; **F**. NC-VEGF siRNA (NC^siRNA^). MSCs were seeded (2.5×10^5^) in 6-well plates 24 hours prior to transfection. The NC^miR^, miR-377, Anti-377, NC^Anti-377^ and VEGF siRNA were added at the final concentration (100 nM for each well) after mixing with DharmaFECT Duo Transfection Reagent (Thermo Scientific) according to the manufacturer’s instructions. 48 hours after transfection, the cells of each group were harvested, followed by PCR and Western blot analysis.

### Dual-Luciferase Reporter Assay

Rat VEGF 3′-UTR (nt1660–3545) was inserted into the Dual-Luciferase reporter vector (pEZX-MT01, Genecopoeia Corp. MD, USA) downstream from the Firefly luciferase (hLuc) reporter gene, and was driven by SV40 Enhancer promoter. In addition, Renilla luciferase (hRLuc) reporter driven by a CMV promoter was cloned into the same vector, serving as the tracking gene and internal control. The dual-reporter vector system enabled transfection-normalization for accurate across-sample comparison. The 293TN cells were assigned into three groups to be transfected with **A.** pEZX-MT01 vector; **B.** pEZX-MT01 vector+NC-miR-377 mimic; **C.** pEZX-MT01 vector+miR-377 mimic. Cell lysates were collected and assayed 48 hours after transfection. Firefly and Renilla luciferase activities were measured using a Dual Luciferase Reporter Assay System kit (Promega Corp. WI, USA) and each transfected well was assayed in triplicate as described [27]. The mutated pEZX-MT01 plasmid containing the mutated VEGF-3′UTR with mutation in the seed region was synthesized using Phusion™ site-directed mutagenesis kit (New England Biolabs. MA, USA) with the following primer, mutated VEGF 3′UTR forward primer 5′-AAGGATAAAATAGACATTGCTATTCTG-3′; reverse primer 5′-AGACTATATACATAAACATATATATATATATATACAC-3′.

### *In Vitro* Tube Formation Assay

HUVECs were purchased from American Type Culture Collection (ATCC) and cultured in endothelial cell growth medium (Cell Application). HUVECs were transiently transfected with **A.** negative control (NC^miR^/NC^Anti^); **B.** miR-377 mimic; **C.** miR-377 inhibitor; **D.** miR-377 inhibitor+VEGF siRNA. After 48 h, *in vitro* tube formation assay was performed with a tube formation assay kit (Chemicon), per the manufacturer’s instructions. Briefly, ECMatrix Solution was thawed on ice for 1∼2 hours, then was mixed with 10×ECMatrixdilutent (v: v = 9∶1). The mix was added to a 96-well tissue culture plate (50 µl/well) and was placed at 37°C for 1 hour to allow the matrix solution to solidify. HUVECs were digested by 0.125% trypsin and were placed (1×10^4^ cells/well) on top of the solidified matrix solution and incubated at 37°C for 18 hours. Cellular network structures were fully developed and photos were taken using an inverted light microscope at 40× magnification. Total capillary tube length and tube branch points were measured using analytical software Image-Pro Plus 6.0 (IPP, Media Cybernetics, Carlsbad, CA). Tube formation was defined as a structure exhibiting a length four times its width. Five independent fields were assessed for each well, and the average number of tubes was calculated [28].

### Lentiviral Overexpression and Suppression of miR-377

Lentiviral vectors (pEZX-MR03) for overexpression and suppression of miR-377 (Null, miR-377, and Anti-377) were purchased from Genecopoeia Corp. (MD, USA) and Applied Biological Materials Inc. (ABM, MC, Canada). Lentiviral particles were produced via transfection of the lentiviral vectors into 293T cells per manufacturer’s protocol.

### *In Vivo* Studies

SD rats (200–250 g) were randomly divided into the following groups to evaluate the direct effects of miR-377 on angiogenesis of MI model: 1.) Sham operated rats had a loose suture placed around the left anterior descending (LAD) coronary artery (Sham group); 2.) Myocardial infarction alone created by LAD ligation (MI group); 3.) MI plus PBS treatment (MI+PBS group); 4.) MI plus MSC^Null^ transplantation (MI+MSC^Null^ group); 5.) MI plus MSC^miR-377^ transplantation (MI+MSC^miR-377^group); 6.) MI plus MSC^Anti-377^ (MI+MSC^Anti-377^ group).

### Surgical Procedures for the LAD Occlusion and MSC Implantation

An MI model was developed in SD rats (200–250 g), as described previously [29]. Briefly, isofluorane anesthesia was induced by spontaneous inhalation. The animals were mechanically ventilated with room air supplemented with oxygen (1.5 L/min) using a rodent ventilator (Model 683; Harvard Apparatus, South Natick, MA). Body temperature was carefully monitored with a probe (Cole Parmer Instrument, Vernon Hill, IL) and was maintained at 37°C throughout the surgical procedure. The heart was exposed by left side limited thoracotomy, and the left anterior descending coronary artery (LAD) was ligated with a 6-0 polyester suture 1 mm from the tip of the normally positioned left auricle. MSCs (30 µl, 2×10^6^) were injected into border area of the left ventricle (LV) wall at 10 minutes after LAD ligation. The chest was closed with 5-0 silk sutures. Approximately 10% of rats succumbed during surgical procedures.

### Immunohistochemical Analysis

Immunohistochemical studies were performed on heart tissue at 4 weeks after cell implantation. Heart tissue sections were harvested, fixed in 10% Formalin, and sectioned at 5-µm thickness. The cardiac troponin T (cTnT) antibody (Thermo Scientific) was used to identify cardiomyocytes, while 4′, 6-diamino-2-phenyindole (DAPI, Sigma) was used to identify nuclei. Von Willebrand Factor (vWF, DAKO, Agilent Technologies) rabbit polyclonal antibody (Santa Cruz Biotechnology) and α-smooth muscle actin (SMA) mouse monoclonal antibody (Sigma) were used to assess capillary and vascular density. Fluorescence labeled secondary antibodies (Jackson Immuno Research Laboratories or Molecular Probes) were used following these primary antibodies. Fluorescent imaging was performed with an Olympus BX41 microscope (Olympus America Inc., Melville, NY, U.S.A.) equipped with epiflourescence attachment, and images were recorded using a digital camera with MagnaFire 2.1 software.

### Measurement of Infarct Size

Fixed hearts were embedded in paraffin, and sections from apex, mid-LV, and base were stained with Masson’s Trichrome. An Olympus BX41 camera was used to obtain images of LV area on each slide using MagnaFire (Olympus) software. Fibrosis and total LV area of each image were measured using Image J software, and the percentage of the fibrotic area was calculated as shown: (fibrosis area/total LV area) ×100, as previously described [9].

### Assessment of Heart Function

Heart function was assessed by transthoracic echocardiography, which was performed at 4 weeks after MI using iE33 Ultrasound System (Phillips) with a 15-MHz probe. After rats were anesthetized with pentobarbital sodium (40 mg/kg) by intraperitoneal injection, hearts were imaged two-dimensionally in long-axis view at the level of the greatest LV diameter. This view was used to position the M-mode cursor perpendicular to the LV anterior and posterior walls. The LV end-diastolic diameters (LVDd) and LV end-systolic diameters (LVDs) were measured from M-mode recordings according to the leading-edge method. LV parameters were obtained from two-dimensional images. LV ejection fraction (EF) was calculated as: EF (%) = [(LVDd)^ 3^- (LVDs)^ 3^]/(LVDd)^ 3^×100. Fractional shortening (FS) was measured using the equation FS (%) = [(LVDd − LVDs)/LVDd] ×100. All echocardiographic measurements were averaged from at least three separate cardiac cycles.

### Statistical Analysis

Experiments were performed in quadruplicate and repeated at least three times. Data are expressed as means ± SE. Statistical significance was assessed by one-way ANOVA followed by Bonferroni/Dunn testing. *P*<0.05 was considered statistically significant.

## Results

### miRNAs Profiling in Rat MSCs under Hypoxia and Normoxia

**Figure 1:**
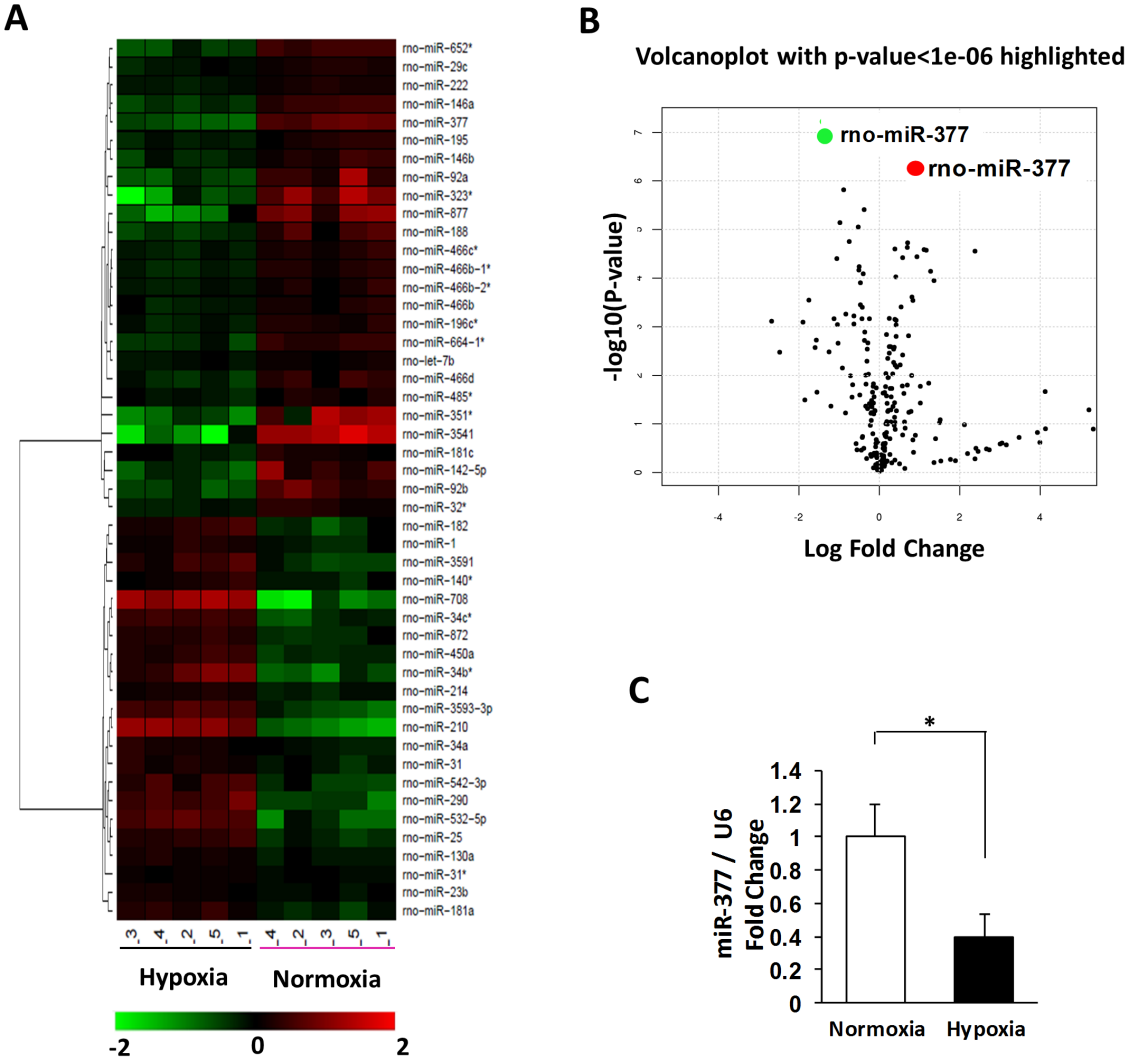
miRNA expression profile is determined in hypoxia-treated mesenchymal stem cells (MSCs). (**A**): A heat map of all dys-regulated miRNAs in hypoxia-treated rat MSCs miRNA microarray. All of the miRNA array raw data are available in the online supplemental Table (n = 5 independent experiments). (**B**): Microarray data are summarized by volcano plot graph, which displays both fold-change and t-test criteria (log odds). MiR-377 and miR-210 are the most significantly dysregulated miRs in hypoxia-treated rat MSCs compared to normoxia-treated MSCs (the green stands for down-regulation, and the red stands for upregulation). (**C**): Alterations in expression levels of miR-377 was validated by qPCR (normalized to control U6, n = 5, p<0.05).

miR microarray analysis was used to examine the potential mechanisms underlying MSC-induced myocardial angiogenesis in response to hypoxia. Among 679 miR probes, a total of 48 miRs were identified that were differentially expressed in hypoxia-treated MSCs as compared to normoxia-treated samples (n = 5, p<0.01, Fig. 1A). When the signal density of miRs was cut off by a value of 100, a group of 13 miRs were significantly up-regulated including miR-210, -25, -450a, -130a, -3593-3p, -34c*, -214, -181a, -23b, -34a, -31, -31*, and -140*; whereas 20 miRs (miR-377, -146a, -222, -652*, -466b-1*, -664-1*, -196c*, -466c*, -29c, -32*, -195, -466b, -188, -146b, -92a, let-7b, -466b-2*, -466d, -485*, and -181c) were significantly down-regulated in MSCs under hypoxic conditions. miR-210 was the most significantly increased miR in hypoxia-treated MSCs (Fig. 1B), a finding consistent with previous observations in tumor cells, endothelial cells, and cardiomyocytes in responsive to hypoxia [15], [30], [31]. miR-377 was the most down-regulated in MSCs upon hypoxia treatment (Fig. 1B). This finding was further validated by real-time stem-loop PCR (Fig. 1C). While miR-210 is well recognized to have pro-angiogenic property *in vivo* and *in vitro* [15], [17], it is unknown whether hypoxia-responsive miR-377 also regulates angiogenesis. Moreover, it is also unknown whether alteration of miR-377 expression affects MSC-induced angiogenesis in ischemic myocardial tissue. Therefore, determination of the role of miR-377 in myocardial angiogenesis and its associated mechanisms could be of major significance for techniques aimed at regeneration of heart tissue.

### Reduced Expression of miR-377 in HUVECs Promotes Angiogenesis

**Figure 2:**
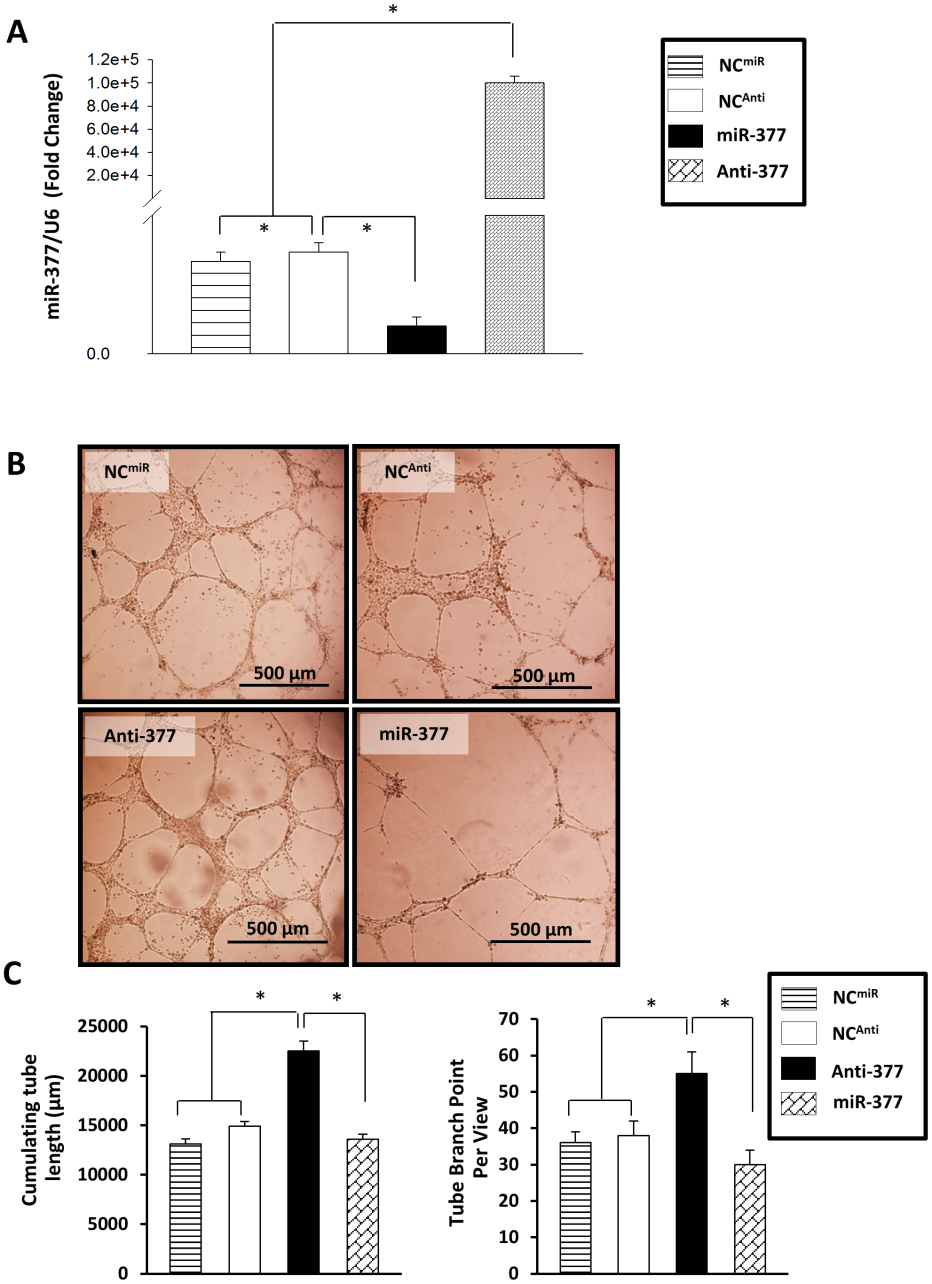
Suppression of miR-377 induced *in vitro* formation of capillary-like structures. (**A**): qPCR analysis after normalization against U6 verified the knockdown of endogenous miR-377 (Anti-377) and overexpression of miR-377 (miR-377) into HUVECs transfected with either miR-377 inhibitor or mimic. (**B**): *In vitro* tube formation assay indicated that knockdown of miR-377 enhanced the formation of capillary-like structures, but this effect was limited by miR-377 overexpression. Scale bars = 500 µm. (**C**): Total capillary tube lengths and tube branch points were measured by analytical software Image-Pro Plus 6.0 (IPP). All values were expressed as means ± SE; n = 6 independent experiments for each group; *P<0.05.

HUVECs were transfected with either miR mimic to overexpress miR-377 or miR inhibitor to specifically knockdown miR-377 (Fig. 2A) in order to determine the significance of miR-377 in angiogenesis, followed by an *in vitro* tube-formation assay using Matrigel-precoated wells. HUVECs transfected with either negative control miR mimic (NC^miR^) or negative control miR inhibitor (NC^Anti^) was used as negative control groups. The negative control (NC^miR^ and NC^Anti^) groups exhibited some tube-like shapes and half-full cellular networks, while the miR-377 mimic group (miR-377 group) revealed less tube-like structures and hardly formed cellular networks (Fig. 2B). However, the miR-377 inhibitor group (Anti-377 group) displayed the formation of full and dense cellular networks (Fig. 2B). The cumulative capillary tube length, measured using IPP software, was reduced by 40±6% in miR-377-HUVECs, whereas it was increased by 54±5% in Anti-377 cells when compared to negative controls (NC^Anti^) (Fig. 2C). No significance was observed between NC^miR^ group and NC^Anti^ group. In addition, the number of tube branch point in miR-377 group (19.5±2.2) was less than that of negative controls (NC^miR^36.0±2.9; NC^Anti^38.0±3.0 respectively), and was significantly increased in Anti-377 group (55.0±3.5) (Fig. 2C).

### MiR-377 Acts Directly at the 3′UTR of VEGF

**Figure 3:**
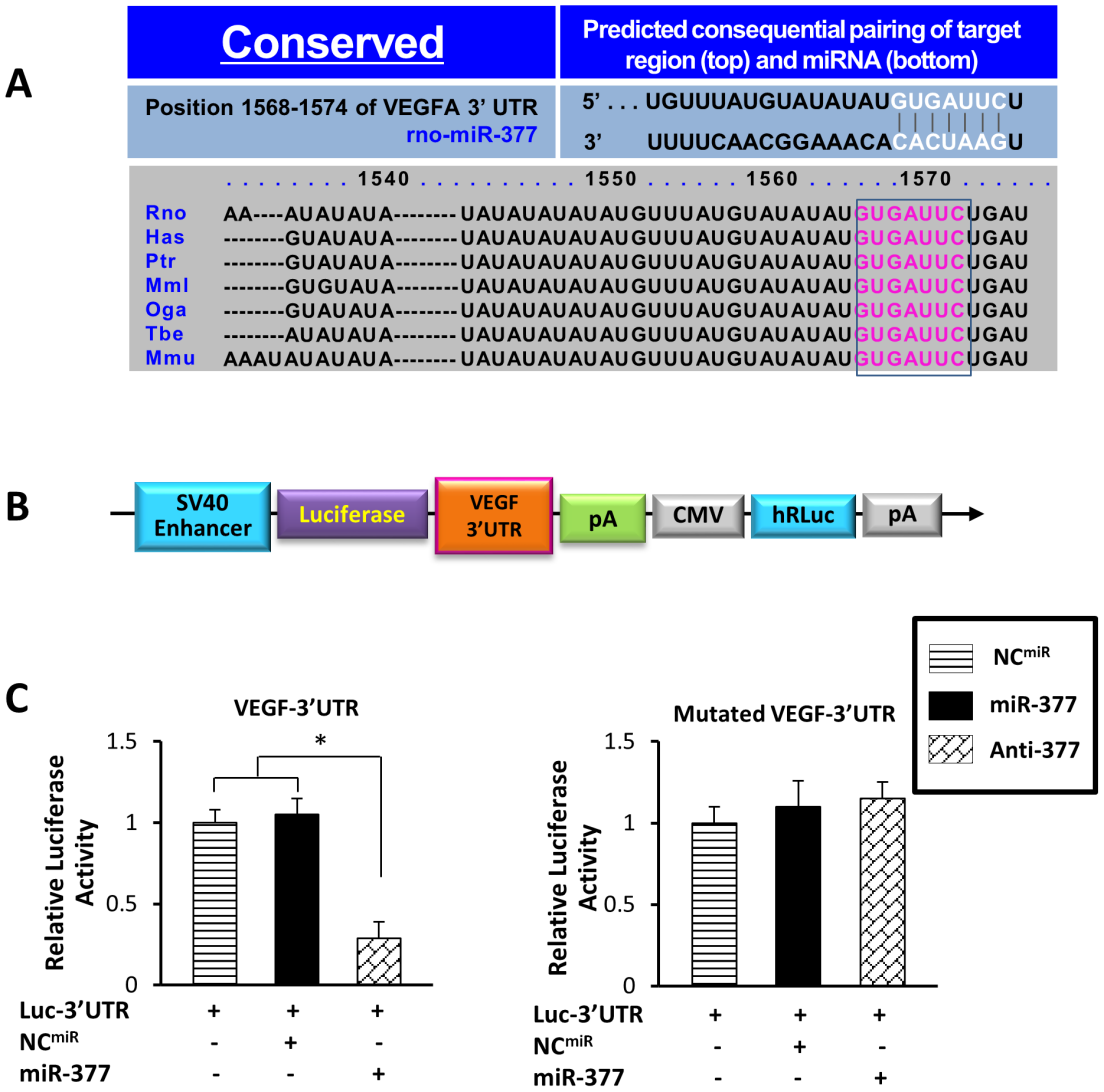
Dual-luciferase reporter assay validates that miR-377 directly targets VEGF. (**A**): Computational miRNA target prediction analysis coincidentally reveals that the fragment 5′-GAAUCAC-3′ of miRNA-377 pairs well with the fragment 5′-GUGAUUC-3′ located at the 1568–1574 nt of VEGF 3′ UTR, which is a highly conserved site (red fonts) in most of mammals (e.g. rat, human, chimpanzee, rhesus, bushbaby, treeshrew, mouse). (**B**): Schematic diagram of Dual-Luciferase reporter vector (pEZX-MT01) carrying the VEGF3′ UTR. (**C**): Quantitative data for dual-luciferase reporter assay results. All values were expressed as means ± SE; n = 6 for each group. *P<0.05 was considered statistically significant.

Computational miRNA target prediction analysis was performed to elucidate the potential mechanism of miR-377 in the regulation of angiogenesis, using TargetScan and miRDB. VEGF-A (usually referred to as VEGF) is listed among the top of assumed targets for rno-miR-377 and the seed sequence of VEGF 3′UTR interacting with rno-miR-377 is highly conserved among the species of rat, human, chimpanzee, rhesus, bushbaby, treeshrew, and mouse (Fig. 3A). HEK293TN cells were transfected with a Dual-Luciferase reporter vector containing the 3′-UTR of VEGF or mutated 3′-UTR of VEGF fused downstream to the Luciferase coding sequence (Fig. 3B) along with miR-377 mimic or a NC (NC^miR^) to validate whether miR-377 directly recognizes the 3′UTR of VEGF. Luciferase activity was repressed by 67% when miR-377 was co-expressed with the VEGF-3′-UTR luciferase reporter vector (Fig. 3C), whereas luciferase activity of mutated VEGF-3′-UTR was not affected. In contrast, transfection with NC did not affect the activity of luciferase.

### MiR-377 Negatively Regulates VEGF Expression in MSCs and ECs

**Figure 4:**
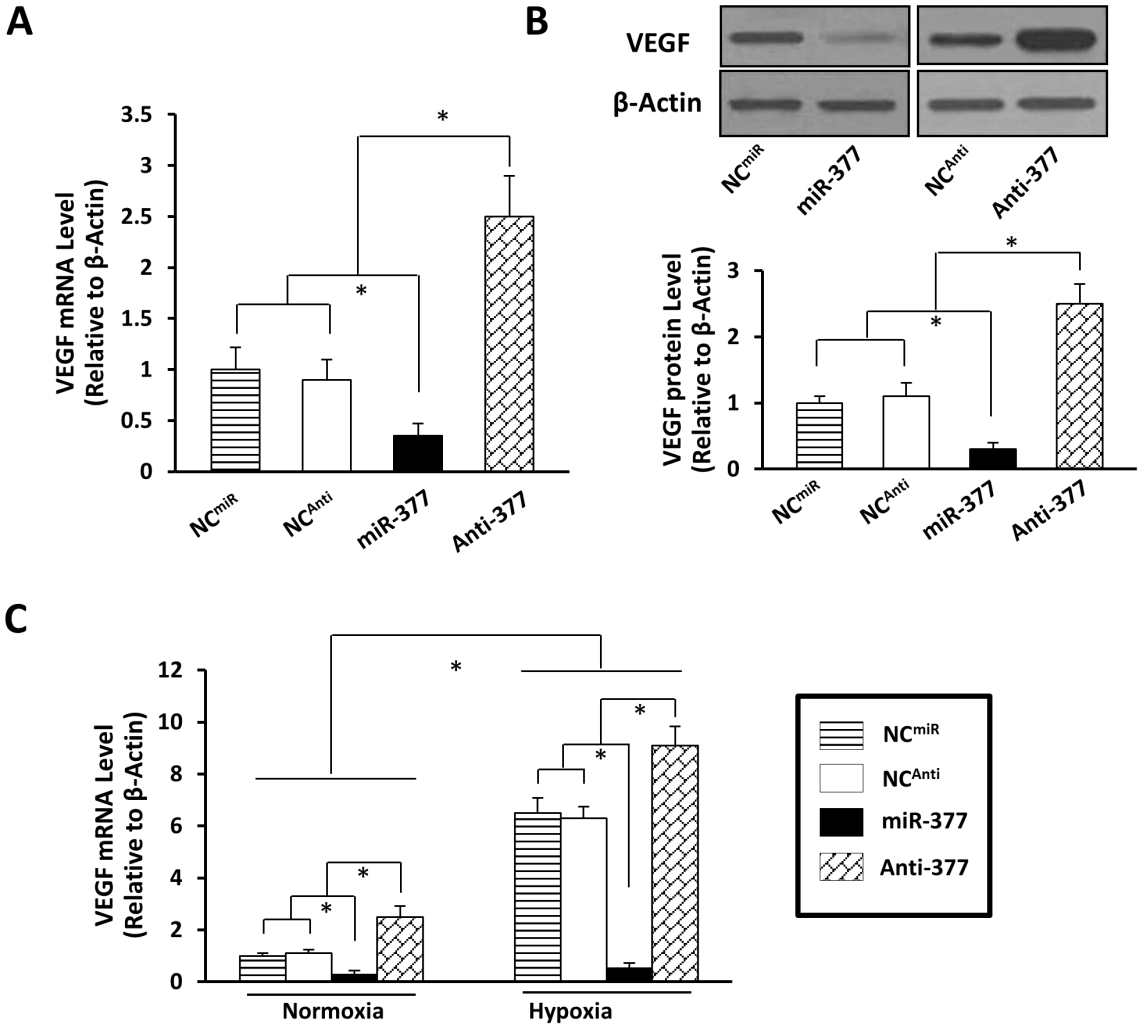
miR-377 directly down regulates the expression of VRGF in MSCs. (**A**): qPCR analysis after normalization against β-actin showed that miR-377 mimic (miR-377) downregulated VEGF mRNA in MSCs, while miR-377 inhibitor (Anti-377) upregulated VEGF mRNA in MSCs. MSCs transfected with a scrambled sequence according to miR-377 mimic and miR-377 inhibitor as negative control (NC^miR^ and NC^Anti^ respectively). (**B**): Western blot assay showed protein level changes of VEGF in MSCs induced by miR-377 mimic (miR-377) and miR-377 inhibitor (Anti-377) as well as its quantitative data. All values were expressed as means ± SE; n = 8 for each group; *P<0.05 was considered statistically significant. (**C**): qPCR analysis after normalization against β-actin showed significant up-regulation of VEGF in hypoxia-treated MSCs in comparison with normoxia-treated MSCs, which was further increased in MSC^Anti-377^, when compared with NC group and miR-377 groups. All values were expressed as means ± SE; n = 8 for each group. *P<0.05 was considered statistically significant.

MSCs were harvested 48 hours after transfection with either NC-miR (NC^miR^), NC-Inhibitor (NC^Anti^), miR-377 mimic (miR-377), or miR-377 inhibitor (Anti-377) to ascertain if miR-377 modulates VEGF expression. Elevation of miR-377 in MSCs suppressed VEGF expression at both mRNA and protein levels (by ∼70%) in Fig. 4A and 4B, which conversely were upregulated by ∼2.5-fold in miR-377-knockdown MSCs (Anti-377) (Fig. 4Aand 4B). In addition, we observed that VEGF was significantly increased in normoxia-treated Anti-377 group as compared with other groups, which further increased in hypoxia-treated Anti-377 group (Fig. 4C). This was well correlative with the reduced expression of miR-377 (Fig. 1C).

### Negative Effects of miR-377 in Angiogenesis are Largely Dependent on VEGF

**Figure 5:**
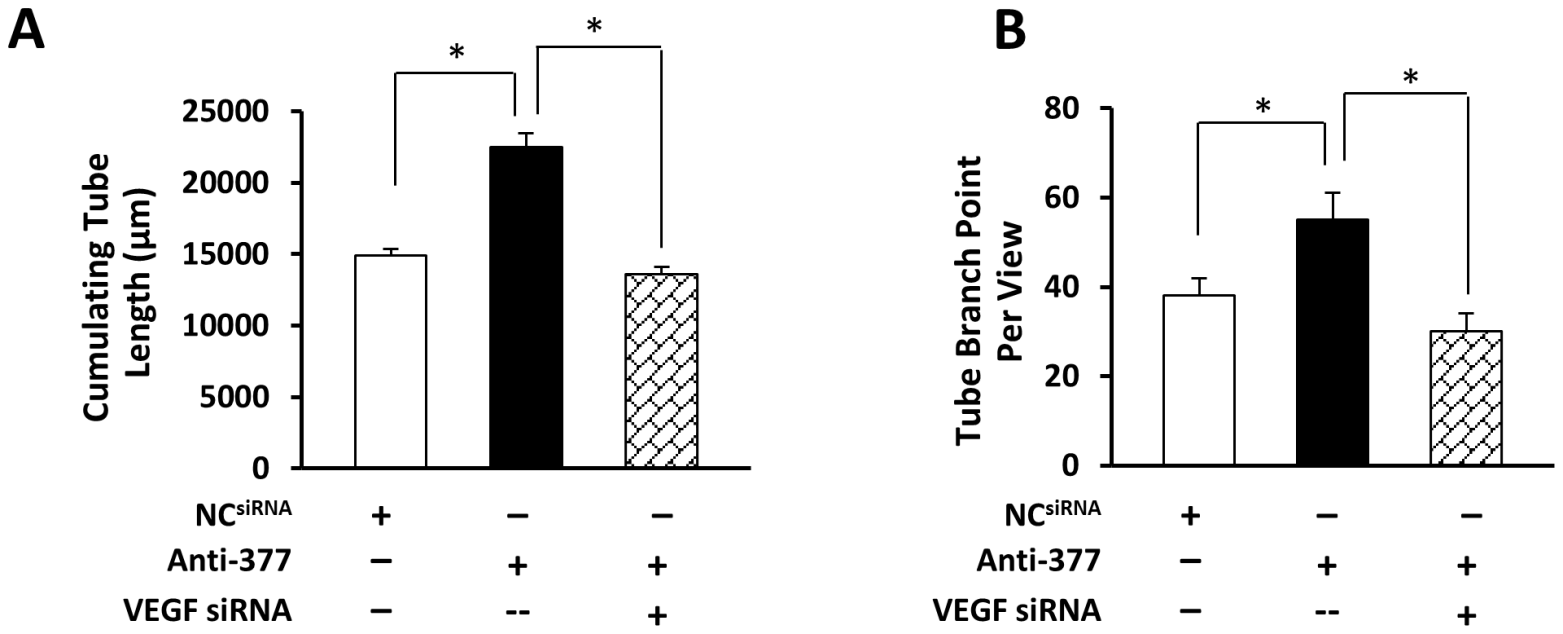
Pro-angiogenic effects elicited by knockdown of miR-377 are largely dependent on VEGF. *In vitro* Tube Formation Assay showed that total capillary tube lengths (**A**) and tube branch points (**B**) were significantly reduced by the VEGF siRNA transfection in miR-377-knockdown HUVECs. All values were expressed as means ± SE; n = 6 for each group; ^*^ *P*<0.05 was considered statistically significant.

It is important to determine whether miR-377 reduction-caused angiogenesis is dependent on VEGF given that VEGF is a target for miR-377. The expression of VEGF was knocked down in miR-377-reduced HUVECs by siRNA, followed by *in vitro* tube formation assay. Similar to previous findings (Fig. 2), it was observed that miR-377-inhibitor-transfected HUVECs had well-developed networks of capillary-like tubes, evidenced by a 1.5-fold increase of cumulative tube length and 1.45- fold increase of tube branch points, compared with NC^miR^ cells. In contrast, HUVECs co-transfected with miR-377 inhibitor+VEGF siRNA exhibited sparse capillary-like structures in which the tube length and the number of tube branch points are similar to NC group (Fig. 5A and 5B). These results indicate that enhanced effects in the formation of tube-like structures induced by knockdown of miR-377 were abolished by inhibition of VEGF expression.

### Suppression of miR-377 in MSCs Enhances Angiogenesis in Ischemic Hearts

**Figure 6:**
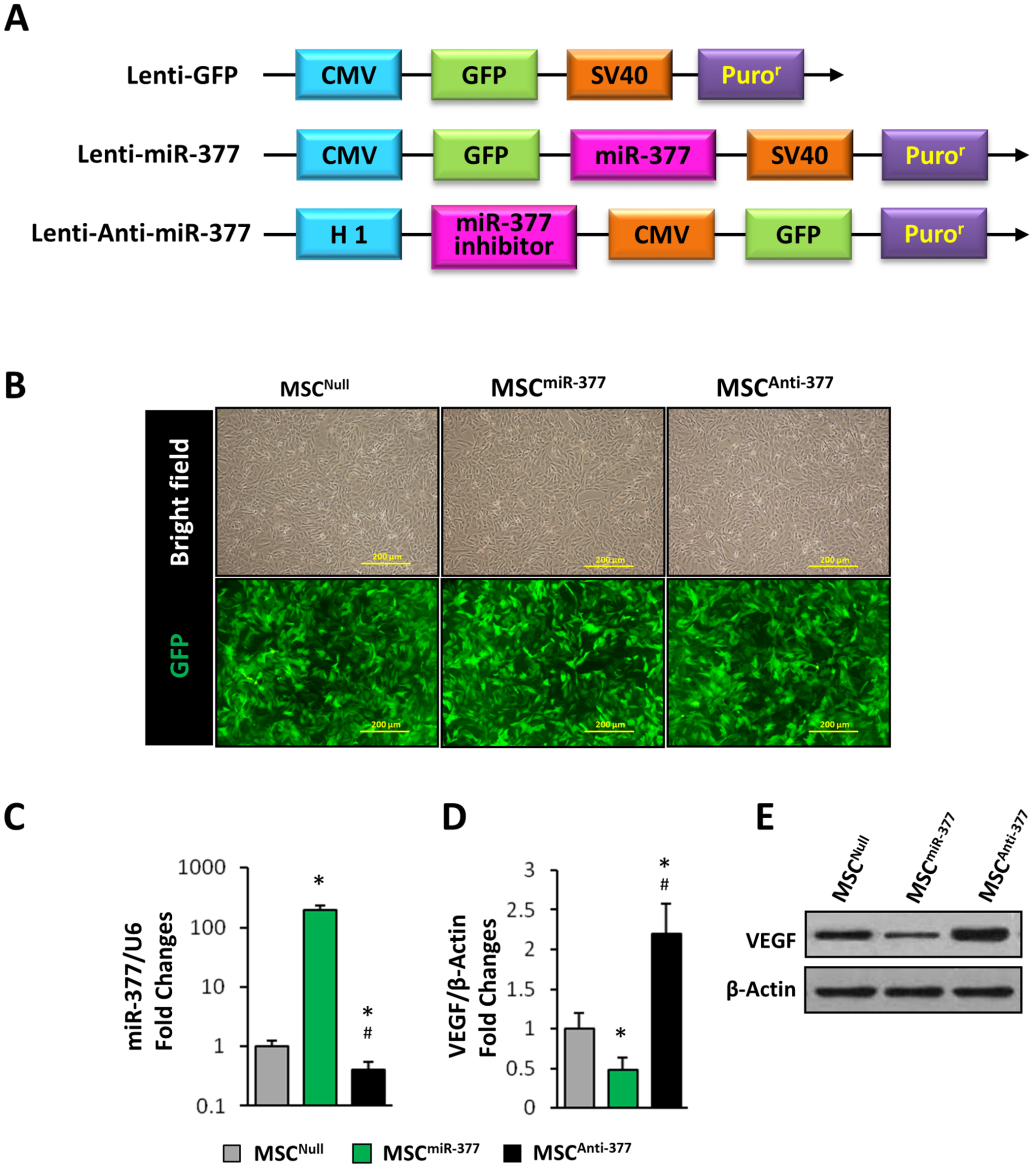
Engineering rat MSCs with lentiviral vectors to overexpress or knockdown of miR-377. (**A**): Schematic diagram of recombinant lentiviral vectors. Lenti-GFP, lentiviral empty vector; Lenti-miR-377, lentiviral miR-377 overexpressing vector; Lenti-Anti-miR-377, lentiviral miR-377 inhibitor expression vector. (**B**): Nearly 100% of MSCs were transfected with Lenti-GFP (MSC^Null^), Lenti-miR-377 (MSC^miR-377^) or Lenti-Off-miR-377 (MSC^Anti-377^) after 72-hour lentiviral infection, as indicated by GFP fluorescence. No morphological changes were found among MSC^Null^, MSC^miR-377^, and MSC^Anti-377^. Scale bars = 200 µm. (**C**): qPCR analysis after normalization against U6 showed that the knockdown of miR-377 in MSC^Anti-377^ and the overexpression of miR-377 in MSC^miR-377^. (**D**): qPCR analysis after normalization against β-Actin. (**E**): Western-blot consistently showed that the expression of VEGF was reduced in MSC^miR-377^ while increased in MSC^Anti-377^, compared with that in MSC^Null^. All values were expressed as means ± SE; n = 6 for each group; ^*^ *P*<0.05 *vs.* MSC^Null^; ^#^ *P*<0.05 *vs.* MSC^miR-377^.

**Figure 7:**
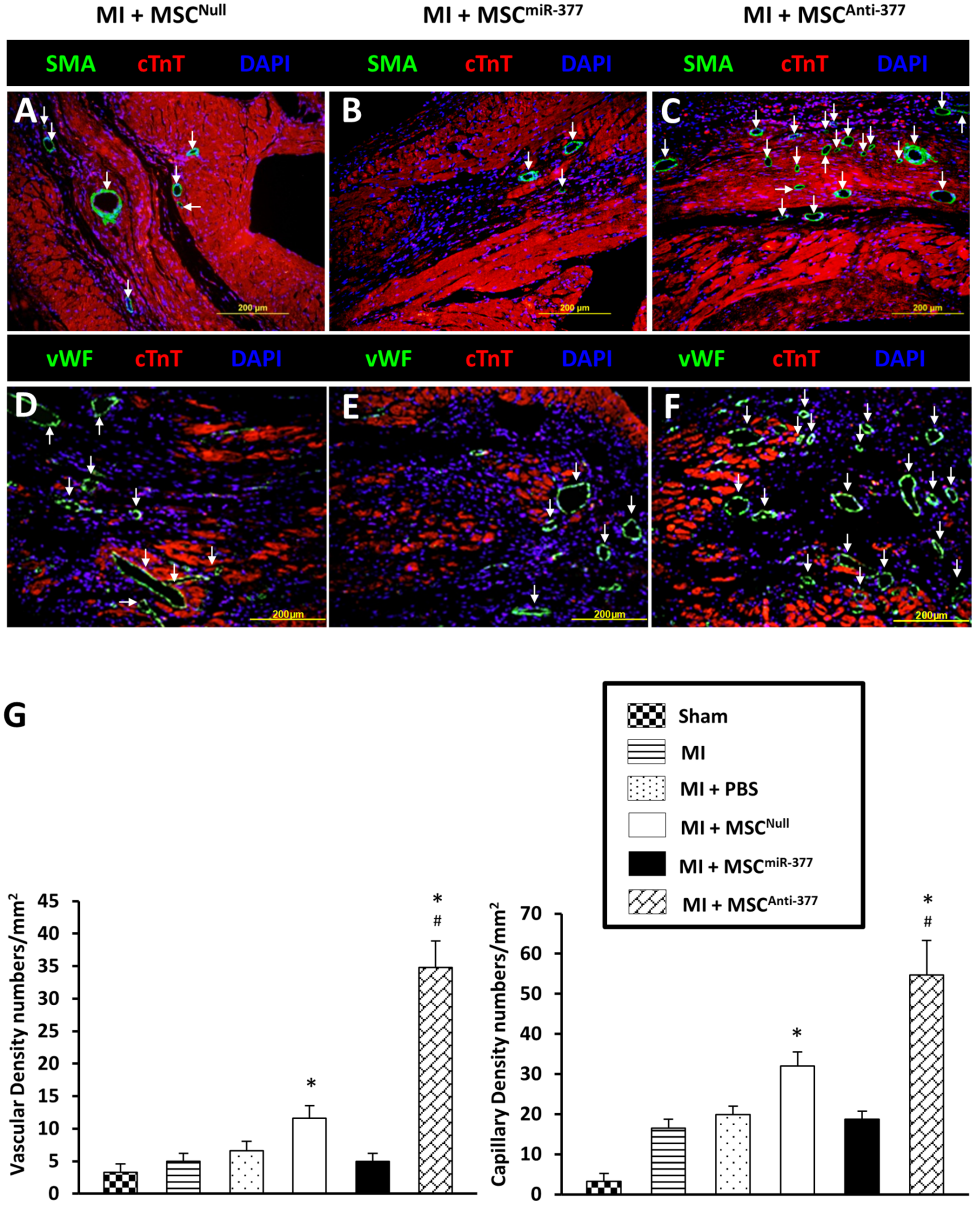
Knockdown of endogenous miR-377 enhances MSC-mediated myocardial angiogenesis in vivo. (**A**): Slice sections of heart samples, collected from MI rats 4 weeks after the injection of miR-engineered MSCs, were triple-stained with troponin I (cTnT) antibody (Ab) (for cardiomyocytes, red), α-smooth muscle actin (SMA) Ab (for vascular cells, green; white arrows) and DAPI (for nuclei, blue). Vascular density was measured in (A) MSC^Null^ group, (**B**) MSC^miR-377^ group, and (**C**) MSC^Anti-377^group. Capillary density was identified by Von Willebrand (vWF) staining (green; white arrows) in (**D**) MSC^Null^ group, (**E**) MSC^miR-377^ group and (**F**) MSC^Anti-377^ group. IPP software was used to quantitatively analyze (**G**) vascular density, and capillary density in different treatment groups. All values were expressed as means ± SE; n = 6 for each group. ^*^ *P*<0.05 *vs.* MI;^ #^ *P*<0.05 *vs.* MI+MSC^Null^.

Rat MSCs were genetically engineered to overexpress or suppress miR-377 using lentiviral transduction (Fig. 6A) to investigate the functional significance of miR-377 in MSC-induced angiogenesis in ischemic hearts. Nearly 100% of MSCs were infected with Lenti-GFP (MSC^Null^), Lenti-miR-377 (MSC^miR-377^), or Lenti-Off-miR-377 (MSC^Anti-377^) after 72-hour transduction, as indicated by GFP fluorescence (Fig. 6B). qPCR confirmed that MSC^Anti-377^ exhibited lower levels of miR-377, whereas MSC^miR-377^ exhibited significantly higher levels of miR-377 than MSC^Null^ (Fig. 6C). Accordingly, qPCR (Fig. 6D) and Western blot (Fig. 6E) showed that VEGF expression was significantly reduced in MSC^miR-377^ when compared with MSC^Null^, whereas it was significantly increased in the MSC^Anti-377^group. 2×10^6^ MSC^Null^, MSC^miR-377^, or MSC^Anti-377^ was then injected into the border zone of ischemic left ventricular (LV) wall at 10 min. after LAD ligation. 4 weeks later, myocardial vascular density was evaluated by α-SMA immunofluorescence staining. Compared with MSC^Null^ group (11.6±1.9/mm^2^; Fig. 7A and G), the MSC^miR-377^ group exhibited a lower vascular density (4.9±1.2/mm^2^; Fig. 7B and G; *p*<0.05), whereas MSC^Anti-377^group displayed a higher vascular density (34. 8±4.1/mm^2^; Fig. 7C and G; *p*<0.05). Capillary density, as determined by vWF immunofluorescence staining, was significantly decreased in MSC^miR-377^-treated hearts, but was significantly increased in MSC^Anti-377^-treated hearts, compared with MSC^Null^ (Fig. 7D–F, Fig. 7G).

### Reduced Expression of MiR-377 in MSCs Limits Fibrosis and Improves Contractile Function in Infarcted Hearts

**Figure 8:**
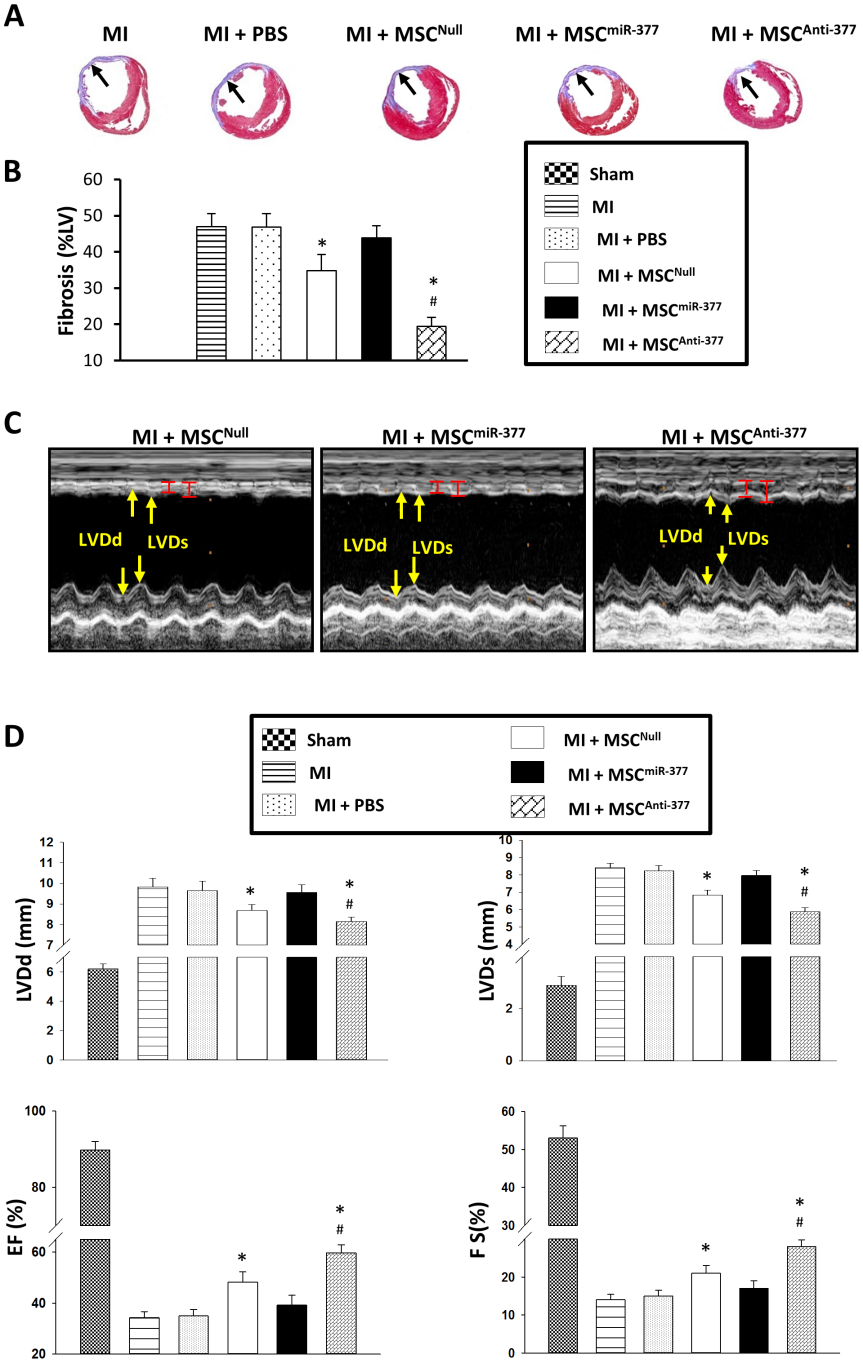
Injection of miR-377-knockdown MSCs into the rat infarcted hearts limits fibrosis and improves cardiac functions. (**A**): 5-µm sections of heart slices, collected from MI rats 4 weeks after the injection of miR-engineered MSCs, were stained with Masson-Trichome. (**B**): Percentage of fibrosis in left ventricle (LV) in various treatments determined with Image J software. All values were expressed as means ± SE; n = 6 for each group. *P<0.05 was considered statistically significant. (**C**): The LV end-diastolic diameters (LVDd, yellow arrow) and LV end-systolic diameters (LVDs, yellow arrow) in MSC^Null^ group, MSC^miR-377^ group and MSC^Anti-377^ group were measured using echocardiographic M-mode recordings. All echocardiographic measurements were averaged from at least 3 separate cardiac cycles. (**D**): Quantitative data of LVDd, LVDs, LV ejection fraction (EF) and fractional shortening (FS) were analyzed and compared among various groups. All values were expressed as means ± SE; n = 6 for each group. ^*^ *P*<0.05 *vs.* MI; ^#^ *P*<0.05 *vs.* MI+MSC^Null^.

Fibrosis area was evaluated using Masson’s Trichrome staining in which normal myocardium was colored red while fiberized myocardium was blue in color due to its inner collagen. The percentage of fibrosis in the left ventricle wall was significantly reduced in the MSC^Anti-377^group, but was increased in MSC^miR-377^-implanted hearts, as compared with MSC^Null^ (Fig. 8A–B).

Echocardiographic measurements showed that cardiac function was significantly improved in MSC^Anti-377^, as evidenced by a shorter LVDd (7.94±0.2 mm) and LVDs (5.875±0.2 mm), a higher ratio of EF (59. 5±2.2%) and FS (26.0±1.8%), compared with MSC^Null^-hearts (8.68±0.3 mm; 6.97±0.3 mm; 48.2±1.9%; 20±1.2%; respectively, *p*<0.05) and MSC^miR-377^ group(9.56±0.38 mm; 7.968±0.3 mm; 39.19±3.9%; 17±1.3%; respectively, *p*<0.05) (Fig. 8C–D). However, no significant differences were noted in these parameters between MI and MI+PBS groups.

## Discussion

Angiogenesis is a key regenerative event in ischemic injured hearts after MI, and VEGF plays an important role in MSC-induced angiogenesis [1]. Given that miRNAs are endogenous regulators of gene expression, it is reasonable to hypothesize that miRNAs may be involved in the regulation of VEGF expression in MSCs. We therefore employed hypoxia, a well-established VEGF inducer, to pre-treat MSCs and determined the miRNA expression profile. The results showed that miR-377 expression was decreased by more than 2-fold in hypoxia-treated MSCs as compared with normoxic condition. By computational miRNA target prediction analysis, we identified VEGF as a potential target of miR-377. Furthermore, both dual-luciferase reporter assay and Western-blotting verified that miR-377 can directly bind with VEGF 3′UTR leading to negatively regulation of its expression.

Accordingly, *in vivo* by transplanting MSCs with genetic overexpression or knockdown of miR-377 in the rat MI hearts, we observed that myocardial angiogenesis was significantly improved in MSC^Anti-377^-treated hearts, whereas it was poor in MSC^miR-377^-treated hearts when comparable to MSC^Null^-injected hearts. It is important to note here, while the degree of fibrosis was less in MSC^Anti-377^-treated myocardium than MSC^Null^-injected group, there were no significant changes in MSC^miR-377^-treated group when compared to controls. Consistent with the alteration of myocardial fibrosis, cardiac function was significantly improved in the MSC^Anti-377^-treatedgroup, but there were no obvious changes in MSC^miR-377^-implanted hearts as compared with MSC^Null^-injected hearts. This may be interpreted that myocardial angiogenesis is reduced in MSC^miR-377^-treated hearts, but not enough to affect MSC-induced beneficial effects on the reduction of fibrosis and improvement of function in infarcted hearts. However, numerous studies have indicated that an increase in myocardial angiogenesis improves contractile function in the infarcted myocardium [10], [32], [33].

It should be noted that VEGF can be regulated either indirectly or directly by different miRNAs in different cells and different diseases [15]–[26]. MiR-210 is a key player of cell response to hypoxia [25] and indirectly up-regulates VEGF-mediated angiogenesis by targeting Ephrin-A3 [15], and enhances MSC-mediated angiogenesis [26]. MiR-145 indirectly down-regulates VEGF in cancer cells to inhibit tumor growth and angiogenesis by targeting p70S6K1, an upstream molecule of VEGF [21]. MiR-10 indirectly down-regulates VEGF-mediated angiogenesis in HUVECs by targeting fms-related tyrosine kinase 1 (FLT1), a cell-surface protein that sequesters VEGF [16]. However, miR-15b, miR-16, miR-20a, miR-20b [17], [18], miR-205 [23] and miR-195 [22] down-regulate angiogenesis by directly targeting VEGF. Such a role has previously only been reported in carcinoma cells, mouse embryonic fibroblast cells, glioma cells, and human hepatocellular carcinoma (HCC) cells. For the first time we demonstrate that miR-377 is responsive to hypoxia and directly targets VEGF in MSCs. Both *in vitro* and *in vivo* evidence presented in this study indicate that knockdown of endogenous miR-377 enhances MSC-mediated angiogenesis and recovery of cardiac function in infarcted myocardium. Therefore, our study indicates that miR-377 may be a novel therapy target for treatment of ischemic heart disease.

## Conclusion

In conclusion, our study indicates thathypoxia-reducedmiR-377 directly targets VEGF, and knockdown of endogenous miR-377 promotes MSC transplantation-induced angiogenesis and subsequent heart function improvement post MI. These data may suggest a new therapeutic strategy for ischemic heart disease treatment in the future.
